# Association between postoperative cough and thyroidectomy: a prospective study

**DOI:** 10.1186/s12885-019-5979-4

**Published:** 2019-07-31

**Authors:** Yao Wu, Qigen Fang, Chunmiao Xu, Hailiang Li

**Affiliations:** 1 Department of Head Neck and Thyroid, Affiliated Cancer Hospital of Zhengzhou University, Henan Cancer Hospital, Zhengzhou, Henan Province People’s Republic of China; 2 Department of Radiology, Affiliated Cancer Hospital of Zhengzhou University, Henan Cancer Hospital, Zhengzhou, Henan Province People’s Republic of China

**Keywords:** Thyroidectomy, Cough, Postoperative complication, Leicester cough questionnaire

## Abstract

**Background:**

This study sought to determine whether thyroidectomy is associated with self-reported cough through a prospective analysis.

**Methods:**

Patients undergoing unilateral thyroidectomy were prospectively enrolled. The control group was selected to avoid the effect of general anaesthesia. The experimental group consisted of 300 patients (202 females and 98 males) who underwent thyroidectomy, with a mean age of 48.6 years, and the control group consisted of 103 patients (53 females and 50 males) who underwent other head and neck operations, with a mean age of 50.3 years. All patients were required to complete the Leicester Cough Questionnaire (LCQ) preoperatively and 2 weeks postoperatively.

**Results:**

The postoperative mean total LCQ scores in the experimental and control groups were 17.9 ± 5.0 and 19.8 ± 4.2, respectively; the difference was significant (*p* = 0.014). Adverse event analysis showed that patients in the experimental group scored significantly worse on items for chest or stomach pains, phlegm, feeling in control of coughing, sleep disturbances, coughing bouts, frustration, and feeling fed up with coughing. In the analysis of the three LCQ domains, a significant difference was noted in the physical domain between the two groups (*p* < 0.001). In the one-way analysis of variance, the factors of sex and anaesthesia time were associated with the postoperative LCQ score; in further multifactor analysis of variance, only the factor of sex was significantly related to the postoperative LCQ score.

**Conclusion:**

Thyroidectomy may be associated with postoperative cough, and a higher number of female patients complained of cough and related symptoms.

## Background

A large proportion of the global increase in thyroid cancer is thought to be due to an actual increase in the incidence of papillary thyroid cancer and/or increased detection of the disease [1]. Clinical manifestations are usually minimal or absent, but sometimes patients might complain of hoarseness, difficulty in swallowing, or difficulty in breathing. Surgery is the treatment of choice for head and neck surgeons. When performed by experienced surgeons, thyroidectomy is regarded as a safe and effective surgical option for selected patients with low risks of permanent vocal distortion, swallowing difficulties, and hypocalcaemia [2, 3], but the symptoms of postoperative nausea and vomiting, transient throat pain, local sensory disturbance, and prolonged swallowing time when taking medications are all common after thyroid surgery. Although the main trunk and branches of the recurrent laryngeal nerve are routinely dissected during surgery, sometimes small branches cannot be protected during central lymph node dissection [4]. Thus, the tracheal branches are sometimes inadvertently resected, although excision of these nerves has unclear effects on clinical symptoms. In our cancer centre, we note that patients who have undergone thyroidectomy often have more severe cough than those who have undergone other operations, and we found this phenomenon to be quite worrisome.

The Leicester Cough Questionnaire (LCQ) was developed by Birring et al. [5] and is a valid and reliable health status measure for adults with cough. Application of the questionnaire has been indicated to be reliable in numerous studies [6–8]. Therefore, our goal was to analyse whether thyroidectomy induces self-reported cough using a prospective analysis.

## Patients and methods

The institutional research committee of the Affiliated Cancer Hospital of Zhengzhou University approved our study (approval number: HNZZ20150079), and all participants provided written informed consent. The study was conducted in accordance with the Helsinki Declaration.

From April 2016 to December 2016, patients undergoing unilateral thyroidectomy and central node dissection due to thyroid cancer were prospectively enrolled. Patients with chronic cough were excluded. To remove the interference of general anaesthesia, patients undergoing neck dissection, oral cavity surgery and throat surgery, paediatric patients, and patients with tumour invasion of the recurrent laryngeal nerve were excluded; patients undergoing other head and neck operations were enrolled as the control group. Smokers were defined as patients who smoked at diagnosis or had stopped smoking within the past 1 year [9]. All enrolled patients were required to complete the LCQ preoperatively and at 2 weeks postoperatively.

A total of 403 patients were enrolled. The experimental group comprised 300 patients (202 females and 98 males), with a mean age of 48.6 (range: 22–78) years. A total of 145 patients underwent left thyroid lobe excision, while the rest underwent right thyroid lobe excision; the mean general anaesthesia time was 1.6 (range: 0.8–2.5) h. Nine patients in the experimental group were smokers. The control group comprised 103 patients (53 females and 50 males), with a mean age of 50.3 (range: 18–85) years. In total, 63 patients underwent superficial parotidectomy, and 40 underwent submandibular gland excision; the mean general anaesthesia time was 1.5 (0.5–3) h. Four patients in the control group were smokers. No significant differences were noted in terms of age, smoking status, or general anaesthesia time between the two groups (all *p* > 0.05), but there were more female patients in the experimental group (*p* = 0.004).

The LCQ questionnaire was originally developed in those with chronic cough, but its validity and responsiveness have been demonstrated in the assessment of acute cough or postoperative cough [6, 10, 11]. The LCQ is designed for self-administration and takes less than 5 min to complete. The questionnaire consists of 19 items, and each item represents an adverse event caused by cough. A 7-point Likert scale was used for scoring the responses. These 19 items were divided into three domains that consider the physical impacts (such as the effect of cough on sputum production, stomach pain and chest pain), the psychological impacts (such as the effect of cough on embarrassment/anxiety), and the social impacts (such as cough interfering with the patient’s job/daily life and enjoyment of life). Three domain scores and a total score are calculated on the LCQ, with domain scores ranging from 1 to 7 and total scores ranging from 3 to 21; a higher score reflects better health status [12].

For sample size calculation, we utilized data from our pre-experiment of 30 patients undergoing unilateral thyroidectomy and central node dissection, which showed a mean total score of 18.0 (standard deviation: 5.2). Therefore, we defined the mean total score in the experimental group as 18.0 with a standard deviation of 5.0. With regard to the control group, no similar literature was available for reference, and because general anaesthesia might be slightly responsible for postoperative cough, and we speculated that control patients would have better total scores. Therefore, we defined the mean total score in the control group as 20.0 with a standard deviation of 5.0. Finally, assuming a two-tailed type 1 error rate of 5%, a sample size of 198 patients in the experimental group and 66 subjects in the control group were required to give greater than 80% power to identify a significant difference between the two groups.

General information was compared using Student’s t-test. Comparisons of variables between the two groups were made using unpaired t-tests, while the difference in sex distribution was examined using chi-square analysis. Relationships between general variables and the postoperative LCQ score were first analysed using one-way analysis of variance and then by multifactor analysis of variance. All statistical analyses were performed with SPSS 20.0. A value of *p* < 0.05 was considered significant.

## Results

The preoperative mean total LCQ score was 21 in both the experimental and control groups, and there was no significant difference (*p* > 0.05). The postoperative mean total LCQ scores in the experimental and control groups were 17.9 ± 5.0 and 19.8 ± 4.2, respectively; the difference was significant (*p* = 0.014). Furthermore, adverse event analysis showed that patients in the experimental group scored significantly worse on the items for chest or stomach pain, phlegm, feeling in control of coughing, sleep disturbance, coughing bouts, frustration, and feeling fed up with coughing (Table 1). In the analysis of the three LCQ domains, there was a significant difference in the physical domain between the two groups (*p* < 0.001) (Table 2).

**Table 1:** Comparison on scores of LCQ between experimental group and control group

Adverse events	Experimental group (*n* = 300)	Control group (*n* = 103)	p*
Chest or stomach pains	5.3 ± 1.6	6.8 ± 0.8	< 0.001
Phlegm	4.6 ± 2.5	6.7 ± 0.5	< 0.001
Tiredness	6.5 ± 2.3	6.3 ± 1.6	–
Controlled by cough	3.2 ± 3.1	6.4 ± 1.3	< 0.001
Embarrassment	6.4 ± 1.6	6.6 ± 1.4	–
Anxiety	5.8 ± 2.6	6.1 ± 0.9	–
Interference with job or other daily tasks	6.6 ± 1.8	6.7 ± 1.7	–
Interference with overall enjoyment	6.7 ± 2.0	6.5 ± 1.9	–
Cough by exposure of paints or fumes	6.3 ± 1.3	6.1 ± 1.1	–
Disturbance of sleep	3.3 ± 1.6	6.7 ± 0.7	< 0.001
Coughing bouts	4.6 ± 1.2	6.6 ± 0.9	< 0.001
Frustration	4.2 ± 1.3	6.1 ± 1.2	< 0.001
Feed up	3.6 ± 2.4	6.7 ± 0.9	< 0.001
Hoarse voice	6.8 ± 1.0	6.9 ± 0.7	–
Full of energy	6.9 ± 0.5	6.7 ± 1.1	–
Worry about serious diseases	6.6 ± 0.5	6.8 ± 1.2	–
Concerned the others’ feeling about your cough	6.8 ± 1.6	6.8 ± 0.8	–
Interrupted conversation or telephone calls	6.3 ± 1.0	6.7 ± 1.7	–
Annoying partner, family or friends	6.3 ± 1.8	6.5 ± 2.2	–

*Not significant

**Table 2:** Comparison on scores of physical, psychological and social items between experimental and control groups

Domains	Experimental (*n* = 300)	Control (*n* = 103)	p*
Physical	4.5 ± 1.4	6.6 ± 1.1	< 0.001
Psychological	6.8 ± 1.9	6.6 ± 1.2	–
Social	6.6 ± 1.7	6.6 ± 1.9	–

*Not significant

To identify associations between the variables and the postoperative LCQ score in the experimental group, one-way analysis of variance determined the factors of sex (*p* = 0.024) and anaesthesia time (*p* = 0.041) to be significantly associated with the postoperative LCQ score. No apparent relationships among age, lobe excision side, or smoking status with the postoperative LCQ score were noted (all *p* > 0.05). The mean postoperative LCQ scores were 17.1 and 18.8 for female and male patients in the experimental group, respectively. In the further multifactor analysis of variance, only the factor of sex (*p* = 0.011) was significantly related to the postoperative LCQ score.

## Discussion

Cough occurs frequently following surgical procedures, and its exact cause remains unclear [13, 14]. Several authors have reported that a considerable number of patients have complaints of cough after lung and abdominal surgery [15, 16]. However, few authors have paid close attention to the relationship between cough and head and neck surgery. To our knowledge, the present study is the first to report that the mean total LCQ score was significantly worse in patients who underwent thyroidectomy than in those who underwent other head and neck operations. The control group was designed to avoid the negative interference of general anaesthesia and tracheal intubation, which also cause postoperative cough. In the control group, no patients underwent surgery involving the vagus nerve, trachea, hypopharynx, or oral cavity. Therefore, these findings suggest that thyroidectomy induces self-reported cough, with high reliability, and this knowledge may provide the impetus for better communication with patients during the treatment.

Preservation of the laryngeal nerves is a major concern during thyroid surgery. Manipulation and/or injury of the vagus nerve can impact the functionality and quality of life of patients, with resulting motor and sensory alterations, such as deficits in laryngeal sensitivity, restrictions on acute frequency modulation, and compromised vocal fold mobility. Most previous studies have focused on the analysis of subjective, nonspecific upper aerodigestive symptoms (UADS). A paper published by Pereira et al. [17] noted that the prevalence of UADS was similar before cholecystectomy (15%) and thyroidectomy (13%), but the postoperative prevalence of UADS was higher among patients who underwent thyroidectomy, including higher prevalence of sensation of strangulation (22% vs 0%), nonspecific voice changes (28% vs 3%), and impaired swallowing (15% vs 3%). Moreover, sensation of strangulation was associated with dysphagia and voice changes. In a similar paper by Silva et al. [18], 100 patients who underwent thyroidectomy with intraoperative neuromonitoring (IONM) were designated the neuromonitored group (NMG), and 208 patients who underwent thyroidectomy without IONM were designated as the control group. The authors noted that the proportion of patients in the control group who reported UADS was 45%, with 33.6% of those patients reporting swallowing symptoms and 25.9% reporting voice symptoms. The proportion of patients in the NMG who reported UADS was 39%, with 22% of those patients reporting swallowing symptoms and 27% reporting voice symptoms. Thus, compared with the NMG patients, the control group patients had a greater severity of UADS-related complications and more swallowing symptoms. These findings might be related to injury to the extrinsic perithyroidal neural plexus innervating the pharyngeal and laryngeal structures. Both studies pay attention to the motility function of the nerves to emphasize the significance of nerve preservation.

Few authors have analysed the changes in sensory or related functions after thyroidectomy. Araújo et al. [19] aimed to evaluate the occurrence of sensory symptoms before and after thyroidectomy by using the vocal tract discomfort (VTD) scale questionnaires. The authors noted that higher severity and frequency of throat dryness were reported preoperatively, whereas more severe throat irritation, itching, soreness, and sensation of a lump in the throat were observed postoperatively. There was a reduction in the frequency and severity of throat dryness and choking sensation pre- and postoperatively. Regarding the severity and frequency of VTD sensory symptoms, a reduction in throat dryness was observed at both assessments. Therefore, the authors concluded that reductions in the sensory symptom of choking and in the frequency and severity of throat dryness were postoperatively self-reported by thyroidectomy patients. Jung et al. [20] conducted a questionnaire survey on the role of a humidifier with heated wire circuits on the incidence and severity of postoperative sore throat and cough after thyroidectomy and reported that most patients suffered from mild cough. However, they did not analyse the possible reasons for this phenomenon, and no reliable control group was designed. Moreover, the reliability of the questionnaire was not proven.

As mentioned above, we designed a control group in which there were no operations with anatomical dissection related to the trachea or the vagus nerve in order to exclude the possible effect of general anaesthesia. Another confounding factor was nerve invasion. Patients with tumour invasion of the recurrent laryngeal nerve were excluded from the experimental group. Therefore, it is reasonable to conclude that thyroidectomy may induce self-reported cough.

Notably, more female patients had complaints of cough, which might be because females are more sensitive to external stimuli [21]. Interestingly, there was no significant association between cough and the lobe excision side. We speculated that because there are more trachea branches in the left recurrent laryngeal nerve, excision of the left lobe would be associated with more cough and related symptoms. Further study is thus needed to analyse this interesting finding.

The main limitation of the current study must be acknowledged: first, postoperative cough can be caused by laryngitis or laryngeal trauma related to orotracheal intubation, but we did not routinely examine the larynx using flexible laryngoscopy for patients with cough, as any misclassification would bias our analysis; second, the LCQ questionnaire in the current study only represented the short-term adverse effects of thyroidectomy on cough, and more long-term clinical data are needed to clarify the question.

## Conclusion

In summary, thyroidectomy is associated with postoperative cough, and a higher number of female patients complain of cough and related symptoms.

## Data Availability

All data generated or analysed during this study are included in this published article. The primary data may be obtained from the corresponding author (Email: qigenfang@126.com; Tel: 86371 65587239; Fax: 8637165587998).

## Abbreviations

IONM: Intraoperative neuromonitoring
LCQ: Leicester Cough Questionnaire
NMG: Neuromonitored group
UADS: Upper aerodigestive symptoms
VTD: Vocal tract discomfort
